# Interactions among the mycobiome, bacteriome, inflammation, and diet in people living with HIV

**DOI:** 10.1080/19490976.2022.2089002

**Published:** 2022-06-23

**Authors:** María José Gosalbes, Nuria Jimenéz-Hernandéz, Elena Moreno, Alejandro Artacho, Xavier Pons, Sonia Ruíz-Pérez, Beatriz Navia, Vicente Estrada, Mónica Manzano, Alba Talavera-Rodriguez, Nadia Madrid, Alejandro Vallejo, Laura Luna, José A. Pérez-Molina, Santiago Moreno, Sergio Serrano-Villar

**Affiliations:** a CIBER de Epidemiología y Salud Pública, Madrid, Spain; b Genomics and Health Area, Fundación para el Fomento de la Investigación Sanitaria y Biomédica de la Comunitat Valenciana, Valencia, Spain; c Department of Infectious Diseases, IRYCIS, Hospital Ramón y Cajal, Madrid, Spain; d CIBER de Enfermedades Infecciosas, Madrid, Spain; e Department of Nutrition and Food Science, Universidad Complutense de Madrid, Madrid, Spain; f HIV Unit, Hospital Clínico San Carlos, Madrid, Spain

**Keywords:** Mycobiome, bacteriome, high-throughput sequencing, ITS2, inflammation, diet, HIV

## Abstract

While the intestinal microbiome seems a major driver of persistent immune defects in people with HIV (PWH), little is known about its fungal component, the mycobiome. We assessed the inter-kingdom mycobiome–bacteriome interactions, the impact of diet, and the association with the innate and adaptive immunity in PWH on antiretroviral therapy. We included 24 PWH individuals and 12 healthy controls. We sequenced the Internal Transcribed Spacer 2 amplicons, determined amplicon sequence variants, measured biomarkers of the innate and adaptive immunity in blood and relations with diet. Compared to healthy controls, PWH subjects exhibited a distinct and richer mycobiome and an enrichment for *Debaryomyces hansenii, Candida albicans*, and *Candida parapsilosis*. In PWH, *Candida* and *Pichia* species were strongly correlated with several bacterial genera, including *Faecalibacterium* genus. Regarding the links between the mycobiome and systemic immunology, we found a positive correlation between *Candida* species and the levels of proinflammatory cytokines (sTNF-R2 and IL-17), interleukin 22 (a cytokine implicated in the regulation of mucosal immunity), and CD8+ T cell counts. This suggests an important role of the yeasts in systemic innate and adaptive immune responses. Finally, we identified inter-kingdom interactions implicated in fiber degradation, short-chain fatty acid production, and lipid metabolism, and an effect of vegetable and fiber intake on the mycobiome. Therefore, despite the great differences in abundance and diversity between the bacterial and fungal communities of the gut, we defined the changes associated with HIV, determined several different inter-kingdom associations, and found links between the mycobiome, nutrient metabolism, and systemic immunity.

## Introduction

HIV is a chronic inflammatory disease in which chronic immune dysfunction appears to be affected by the microbiome and contributes to persistent inflammation leading to an excess risk of mortality.^1–5^ However, while the microbiome has emerged as a research specialty in the HIV field, the fungal communities, namely the mycobiome, have received little attention, perhaps because it represents a small (0.1%) and highly variable fraction of the microbiome.^6–8^

For many years, understanding the multiple HIV-associated gut-associated lymphoid tissue defects has been pursued to define new strategies to reduce the long-term consequences of chronic inflammation.^9,10^ Interactions between the immune system and pathogenic fungi are known to occur via C-type lectin receptors (dectin-1) and caspase recruitment domain-containing protein 9 (CARD9).^11–13^ However, the information on the role of the mycobiome in the development and modulation of the immune system remains scarce, and to our knowledge, no studies have characterized the changes in the intestinal mycobiome associated with HIV infection. This delay in our understanding of the mycobiome could be partly explained by the fact that, despite the advance in high-throughput sequencing techniques, the study of the fungal community is still subject to stubborn technical limitations. For example, the mycobiome greatly varies between individuals, may be because its composition depends on multiple factors, being diet the most important and barely considered in the previous literature, which hampers discerning whether the intestinal fungi detected are transient or commensal.

In PWH, some authors have characterized the mycobiome in the oral cavity, the respiratory tract, and the lung.^14–18^ However, there is a gap in knowledge about the role of mycobiome in the gut of PWH, a major site of HIV immunopathogenesis and persistence of chronic immune defects associated with clinical progression. Here, we characterized the intestinal mycobiome in PWH and evaluated its correlations with diet and immunological predictors of clinical progression measured in blood. Our study defines the compositional changes in the fungal communities associated with HIV and unveils new bacteria–fungi interactions linked with diet and systemic immune responses.

## Results

### Mycobiome composition

To assess the mycobiome composition of 24 PWH on ART and 12 healthy controls (HIV-), we performed fungal DNA extractions and Internal Transcribed Spacer 2 (ITS2) amplifications from the fecal samples. The general characteristics of the study population is described in Table 1. The ITS2 amplicon sequencing yielded 5976923 sequences that were clustered in 830 Amplicon Sequence Variants (ASVs), capturing 116 genera and 158 species.

**Table 1.** General characteristics of the study population.

	Control	PWH	Overall
	(N = 12)	(N = 24)	(N = 36)
**Age (years)**			
Median [Min, Max]	48.5 [28.0, 67.0]	46.9 [24.5, 69.4]	47.6 [24.5, 69.4]
**Sex (N, %)**			
Female	7 (58.3%)	2 (8.3%)	9 (25.0%)
Male	5 (41.7%)	22 (91.7%)	27 (75.0%)
**Body Mass Index (kg/height(m^2))**			
Mean (SD)	24.4 (3.65)	25.3 (3.85)	25.0 (3.75)
**Recent antibiotic use**			
No	12 (100%)	24 (100%)	36 (100%)
**Recent antifungal use**			
No	12 (100%)	24 (100%)	36 (100%)
No	12 (100%)	24 (100%)	36 (100%)
***HIV-related variables***			
**Years since HIV diagnosis**			
Median (25th-75th percentile)	-	6.5 (2.9–18.4)	-
**Nadir CD4 + T cells/uL**			
Median (25th-75th percentile)	-	218 (105–294)	-
**CD4 + T cells (counts/uL)**			
Median (25th-75th percentile)	-	555.8 (456, 695)	-
**Baseline HIV RNA (log 10 copies/mL)**			
Median (25th-75th percentile)		4.9 (4.2–5.5)	
**Undetectable HIV RNA (N, %)**		24 (100%)	
**Antiretroviral therapy (N, %)**			
INSTI-based		8 (33.4)	
PI-based		1 (4.1)	
NNRTI-based		15 (62.5)	

Abbreviatures: INSTI, integrase strand-transfer inhibitors; PI, protease inhibitors; NNRTI, non-nucleoside retrotranscriptase inhibitors.

**Figure 1.**
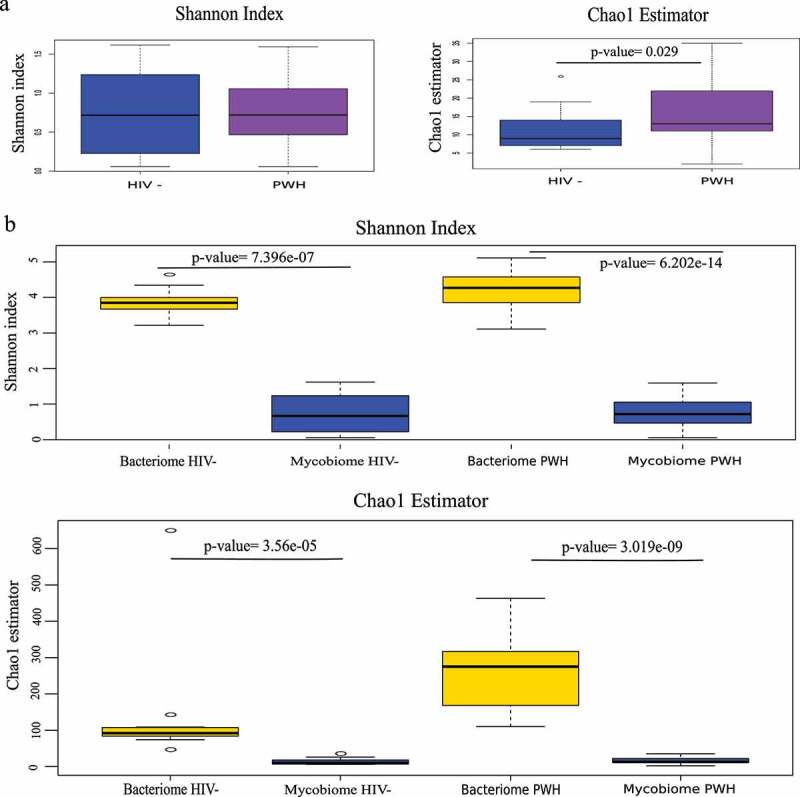
Mycobiome and bacteriome alpha diversity. (a) Shannon diversity index and Chao1 richness estimator of fungal communities from HIV-infected subjects (PWH) and healthy controls (HIV-). (b) Shannon index and Chao1 estimator for mycobiome and bacteriome in PWH and HIV- groups.

**Figure 2.**
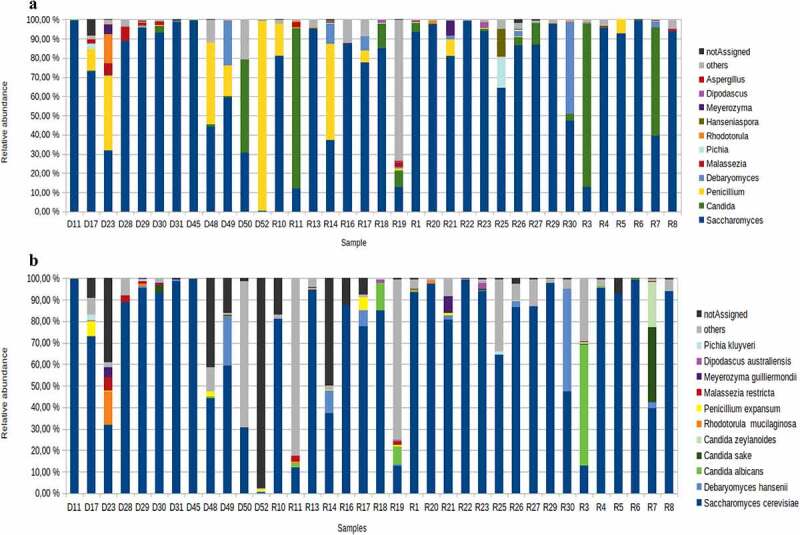
Comparison of fungal composition between PWH individuals and controls. (a) At genus level. (b) At species level. The PWH subjects are labeled with an R and the controls with an D. The genera and species present in at least 25% of the samples have been represented.

The Shannon diversity indexes of the fungal communities from PWH and controls were low in both groups (0.75 vs. 0.79, p-value = ns). However, the richness estimator, Chao1, was significantly higher in PWH (p-value = 0.029) (Figure 1a). We also assessed the composition of the bacterial community (hereafter bacteriome) for the same fecal samples. Figure 1b shows the alpha diversity of the bacteriome and mycobiome in both groups. Overall, Shannon index and Chao1 estimator were significantly higher in the bacteriome than in the mycobiome in both groups, indicating that, regardless of HIV status, the bacteriome is richer and more diverse than the fungal community. Ascomycota was the most abundant phylum in both groups, followed by Basidiomycota phylum which presented higher relative abundance in healthy controls (p-value = ns). Two minor phyla, Mucoromycota, and Chytridiomycota, were specific to PWH. *Saccharomyces* was the most abundant genus across individuals (67.8% in controls vs 73.7% in PWH, p-value = ns), while *Penicillium* was more abundant in controls (17.4% in controls vs 3.8% in PWH, p-value = ns) and *Candida* was more prevalent in PWH (11.4% in controls vs. 4.6% in PWH, p-value = ns) (Fig, 2a). We also observed high inter-individual variability, as indicated, for example, by the finding of *Torulaspora*, mainly *T. delbrueckii*, which was the predominant genus and species in patient R19, or by the finding of minor genera, including *Ustiliago, Starmerella, Kazachstania*, and *Lopistoma* in variable abundance in subjects D48 (11%), D50 (17%), R16 (12%), and R17 (8%). Moreover, differences in the diversity among the samples have been also observed. *Saccharomyces cerevisiae* was the most prevalent yeast and the unique species of *Saccharomyces* genus that was present in all individuals. In contrast, the richness within *Candida* genus was high, including 14 species, being *C. albicans* (33%), *C. zeylanoides* (25%), and *C. sake* (25%) the most dominant. Inter-individual variability was also highlighted in the case of *C. glabrata*, which was predominant in two patients (R11, 81%, and R3, 27%) and *C. quercitrusa* representing 48% in D50. Moreover, five species of *Candida* were specific to PWH, being *Candida parapsilosis* the most prevalent (25%) (Figure 2).

### Mycobiome dysbiosis in people living with HIV

Principal Coordinate Analysis (PCoA) based on Bray-Curtis dissimilarity index at ASV level revealed that the fungal communities of PWH vs. controls differed in terms of beta-diversity (Adonis, p-value = 0.0017) (Figure S1).

**Figure 3.**
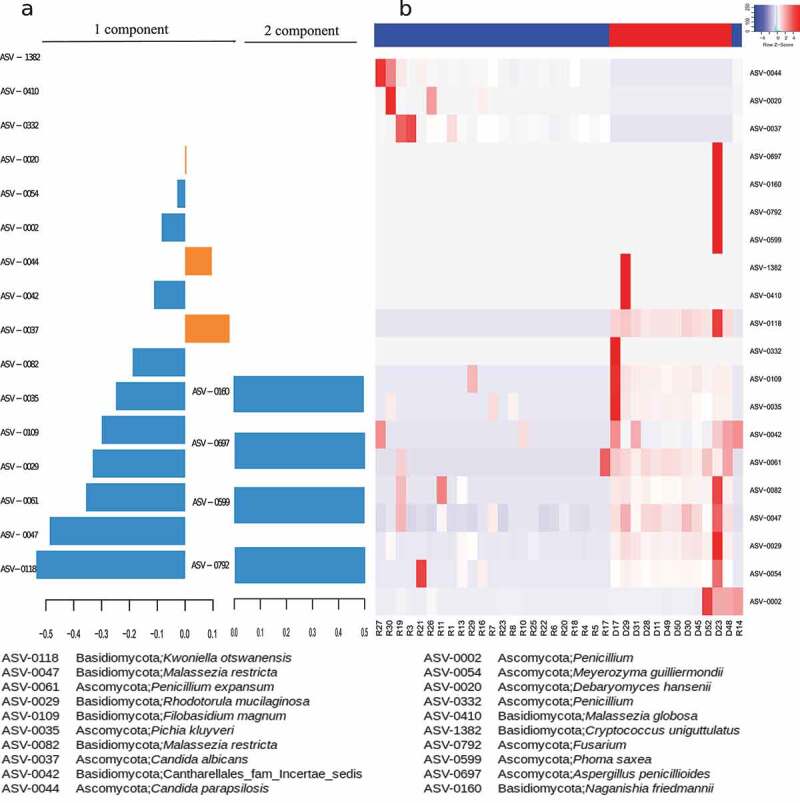
Discriminant analysis of the mycobiome composition between PWH and HIV- groups. (a) Loading values of the selected variables (ASVs) for the first and second components from sPLS model. Orange, ASVs more abundant in PWH group; blue, ASVs more abundant in HIV- group. (b) Heatmap and clustering of the samples according to the abundance of the discriminant ASVs. Blue, samples belonging to PWH group, red samples belonging to HIV- group.

We applied sparse Partial Least Square Discriminant Analysis (sPLS-DA) to further investigate the mycobiome components driving the differences. The performance of our sPLS-DA model displayed an accuracy of 0.955 and we determined 16 and 4 discriminant taxa in component 1 and in component 2, respectively, of which 10 were significantly more abundant in controls and 3 in PWH (Figure 3, Figure S2, Table S1). We found that *Debaryomyces hansenii* (q-value = 3.29e-05), *Candida albicans* (q-value = 3.29e-05) and *Candida parapsilosis* (q-value = 3.29e-05) were the most abundant taxa in PWH, although their discriminant power (loading value in Figure 3a) was only moderate. In controls, the mycobiome was mainly characterized by four mold taxa and five yeasts. Among the molds, *Kwoniella botswanensis* (q-value = 1.35e-06) and *Penicillium expansum* q-value = 9.15e-05) showed the highest discriminant power, while the yeasts *Malassezia restricta* (ASV-0047, q-value = 3.2e-05 and ASV-0082, q-value = 1.36e-04), *Rodothorula mucilaginosa* (q-value = 4.55e-07), *Pichia kluyveri* (q-value = 3.00e-04), and *Meyerozyma guilliermondii* (q-value = 3.89e-04), had remarkable discriminative power in controls. Figure 3b shows the clustering of the samples on the base of the abundance of the differential ASVs determined by sPLS-DA.

### Interactions between mycobiome and bacteriome

**Figure 4.**
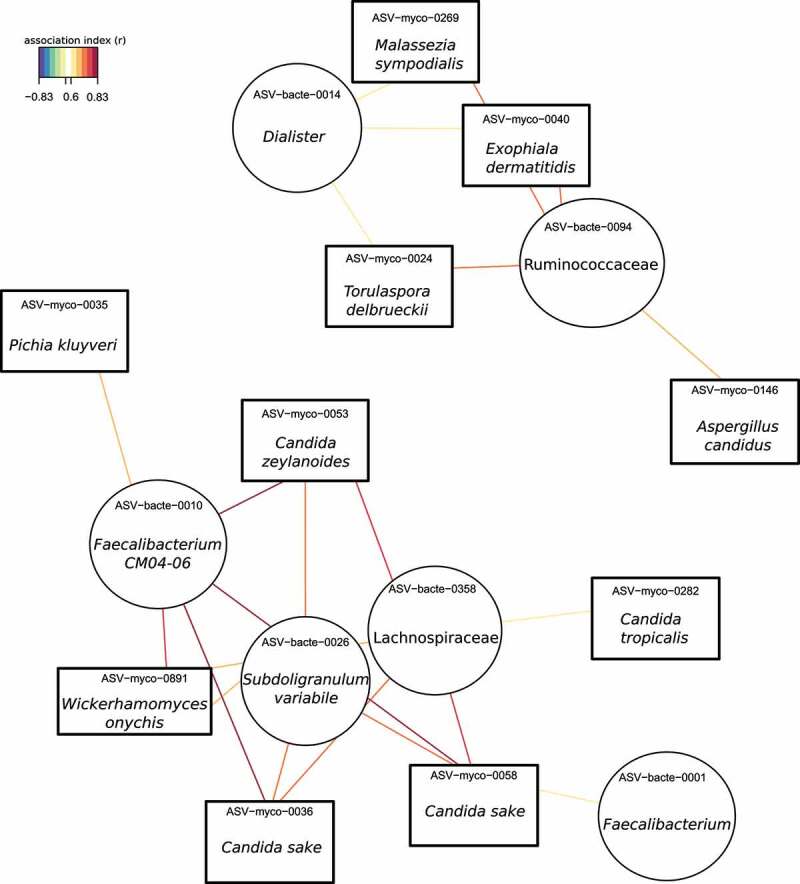
Association network between mycobiome and bacteriome by applying sPLS analysis in PWH group. Association index >0.6.

Following the pattern showed in previous studies,^19–22^ in our analysis the bacteriome showed differences associated to HIV infection (Figure S3). *Prevotella* genus appeared as the major biomarker among PWH, while *Bacteroides* was significantly more abundant in controls (Figure S4). Then, we assessed in PWH and healthy controls the relationships between fungal (hereinafter, ASV-myco) and bacterial (hereinafter, ASV-bacte) taxa applying a multivariate sPLS analysis. In the HIV-associated mycobiome, we detected two clusters with high correlation coefficients, most of them direct correlations (Figure 4, Figure S5, Table S2). We found that *Candida* genus is highly correlated with the bacteriome, being *C. sake* (ASV−myco−0058, ASV−myco−0036) and *C. zeylanoides* presented the strongest correlations (r = 0.83, r = 0.81, and r = 0.80, respectively) with *Faecalibacterium* CM04-06. *P. kluyveri* and *Wickerhamomyces onychis* (also named as *Pichia onychis*) strongly correlated with *Faecalibacterium* genus (r = 0.69 and r = 0.74, respectively). In the other cluster (Figure 4), *Torulospora delbrueckii, Malassezia sympodialis*, and *Exophiala dermatitis* correlated with *Dialister* and a member of Ruminococcaceae family. Moreover, the mold *Aspergillus candidus* also showed a relation with Ruminococcaceae family. The PWH biomarkers showed weak correlations with the bacteriome (Figure S5, Table S2). Interestingly, clear mycobiome–bacteriome interactions were found in healthy controls (Figure S6, Table S3) and six fungal biomarkers strongly correlated with bacterial components, including *Oscillibacter, Subdoligranulum, Lachnoclostridium*, and *Bacteroides uniformi*.

### Combined effects of the mycobiome and bacteriome on systemic markers of immune activation in PWH

**Figure 5.**
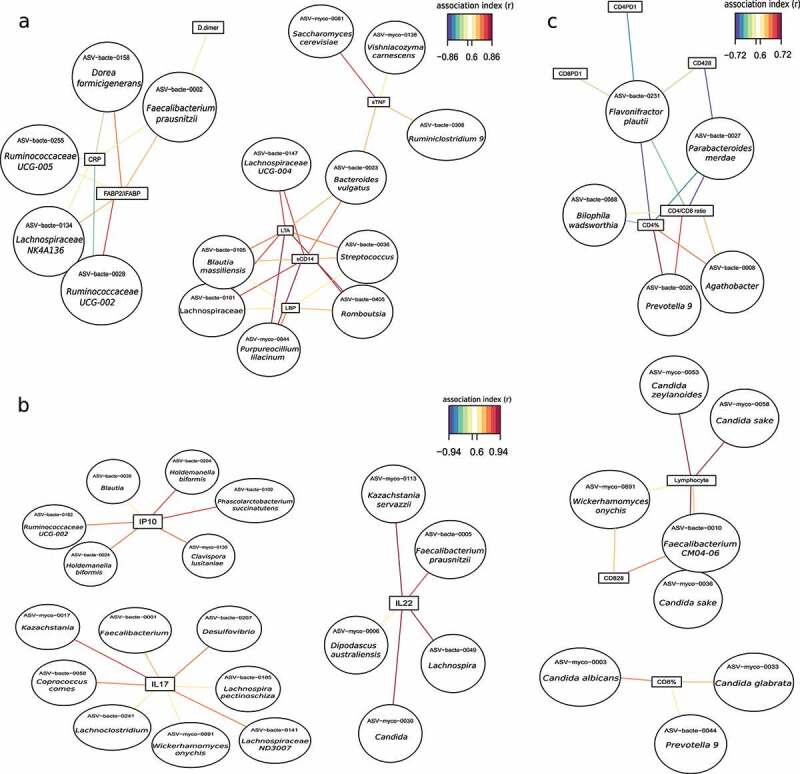
Association network between the microbiome and systemic markers of immune activation. (a) Association network between the microbiome and inflammation markers in the PWH group. Lipoteichoic acid, LTA; Lipopolysaccharide-binding protein, LBP; soluble CD14, sCD14; tumor soluble necrosis factor receptor 2, sTNF-R2; C-reactive protein, CRP; Intestinal fatty acid-binding protein (IFABP). (b) Association network between microbiome and cytokines in PWH group. Interferon-gamma induced protein 10, IP-10; Interleukin-17, IL-17; Interleukin-22, IL-22. (c) Association network between microbiome and T cells in PWH group. CD4 + T cells, CD4%; CD8 + T cells, CD8%; CD4+ CD28- T cells, CD428; CD8+ CD28- T cells, CD828; CD4+ PD1 + T cells, CD4PD1; CD8+ PD1 + T cells, CD8PD1. Association index >0.6.

Although bacteria represent the major component of the gut microbiota, the fungal community could also play a major role on human health. Thus, we considered the microbiome as a whole community integrated by the bacteriome and mycobiome and we assessed the associations with immune activation markers previously shown to independently predict the risk of clinical progression in PWH by using a multivariate sPLS analysis (Table S4).^1–5^ As shown in Figure 5a and Figure S7, the bacterial translocation markers (lipoteichoic acid, LTA and lipopolysaccharide binding protein, LBP) and monocyte activation marker (soluble CD14, sCD14), which are metabolically related, clustered together and directly correlated with *Purpureocillium lilacinum* (r = 0.86, 0.72, 0.82, respectively), a filamentous fungus able to produce opportunistic infections in immunocompetent and immunocompromised hosts.^23^ Also, bacterial members, such as *Blautia, Streptococcus, Romboutsia*, and Lachnospiraceae genus, showed a correlation with these three inflammation markers (Table S5), as previously reported.^21,22^ Other correlations included a direct association between soluble CD163 (sCD163), a biomarker of macrophage activation, with lower association index with *Purpureocillium lilacinum* (r = 0.39), *Romboutsia* (r = 0.44) and *Lachnospiraceae UCG-004* (r = 0.39) although they showed lower association index (Figure S7, Table S5). The prothrombotic marker, D-dimers, and the pro-inflammatory C-reactive protein (CRP), also clustered with a similar association pattern (Figure 5a, Figure S7, Table S5). Interestingly, these biomarkers reflecting activation of the innate immunity negatively correlated with bacterial communities known to elicit immunoregulatory responses and with anti-inflammatory potential, including *Faecalibacterium prausnitzii, Ruminococcaceae UCG-002, Lachnospiraceae NK4A136 group, Dorea formicigenerans* and *Ruminococcaceae UCG-005*.^24^ Two yeasts, *Wishniacozyma carnescens*, rarely reported in the medical literature, and *Saccharomyces cerevisiae*, often used as a probiotic to treat antibiotic-related diarrhea,^25^ directly correlated with D-dimers (r = 0.51, r = 0.52, respectively) and sTNF-R2 (r = 0.61, r = 0.79, respectively) (Figure S7, Table S5). Intestinal fatty-acid binding protein (IFABP) is a biomarker denoting enterocyte barrier integrity that increases following gut damage. We found a direct correlation of IFABP with *Faecalibacterium prausnitzii* (r = 0.66), *Ruminococcaceae UCG-002* (ASV-bacte-0028, r = 0.78), *Lachnospiraceae NK4A136 group* (r = 0.67), *Dorea formicigenerans* (r = 0.75), *Ruminococcaceae UCG-005* (r = 0.65) and *Agathobacter* (r = 0.53), while negatively and weakly correlated with the fungus *Clavispora lusitaniae* and bacteria such as *Phascolarctobacterium succinatutens* and *Holdemanella biformis* (Figure 5a, Figure S7, Table S5). Conversely, interferon-γ-inducible protein 10 (IP-10), a pro-inflammatory cytokine associated with clinical progression in PWH,^26^ directly correlated with the pathogenic yeast *Clavispora lusitaniae* and the bacteria *Phascolarctobacterium succinatutens, Holdemanella biformis, Blautia* and *Ruminococcaceae UCG-002* (Figure 5b). Finally, IL17 and IL22 interleukins directly correlated with the yeasts *Candida, Kazachstania* and *Wikerhamomyces* as well as with bacterial genera such as *Faecalibacterium, Lachnospira, Coprococcus* and *Desulfovibrio* (Figure 5b, Figure S8, Table S5). Finally, we used an sPLS analysis to establish connections between the mycobiome-bacteriome and the adaptive immunity, i.e., total lymphocyte counts and %CD8 + T cells, and immunoactivation markers, as expressed by the %HLADR+CD38+ CD4 + T cells and %CD8+ CD28- T cells. These biomarkers clustered and directly correlated with different species of *Candida* genus and *Wikerhamomyces* (Figure 5c, Figure S9, Table S5). *C. albicans* and *C. glabrata* also correlated with CD8 + T cells (r = 0.67 and r = 0.63, respectively). In addition, we appreciated several associations between bacterial components (*Prevotella* 9, *Agathobacter, Biophila, Flavonifractor, Parabacteroides*) and the systemic immune parameters, such as CD4+ CD28- T cells, CD4 + T cells, and T cell exhaustion markers (CD4+ PD1+, and CD8+ PD1 + T cells) (Figure 5c, Figure S9, Table S5).

### Associations between diet and microbiome in people living with HIV

We performed a comprehensive dietary assessment following the principles previously reported.^27^ The Mediterranean Diet Quality Index (MED-DQI) for the PWH (5.1 ± 2.2) indicated that an average good to medium good-quality diet, with 43% of the total energy intake (TE) originating from carbohydrates, 37% TE from lipids and 17% TE from proteins (Table S6).

**Figure 6.**
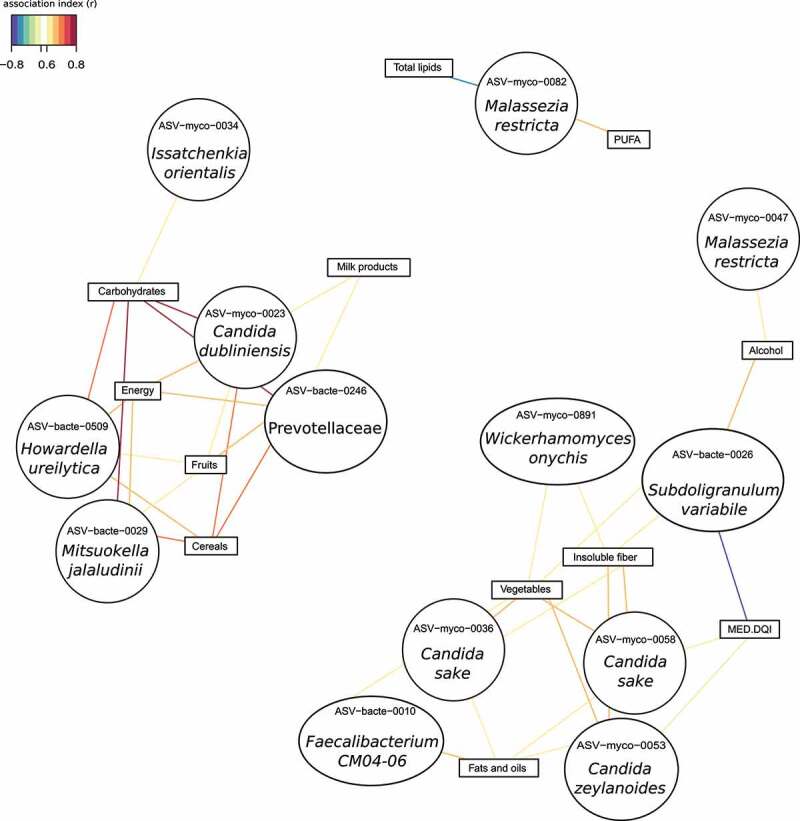
Association network between microbiome and dietary data (food consumption and nutrient intake) in PWH group. Association index >0.6. Mediterranean Diet Quality Index, MED-DQI.

Following the sPLS analysis to integrate the dietary and microbiome information, we found that a cluster including vegetables, total fiber, soluble fiber, and insoluble fiber, was strongly associated with fungal taxa, of which 50% belonged to *Candida* genus (Figure S10, Table S7). Figure 6 showed the associations with an index higher than 0.6. Intriguingly, *Faecalibacterium* and *Subdoligranulum*, both butyrate-producer bacteria that catabolize dietary fiber, positively correlated with the same dietary compounds, suggesting a cause–effect relationship (Figure 6). In fact, *Subdoligranulum variabile* (r = −0.77), *Faecalibacterium CM04-06* (r = −0.51), *Candida sake* (r = -0.6), and *Candida zeylanoides* (r = −0.6) correlates negatively with MED-DQI index for which a lower score indicates a better-quality diet. In contrast, *Saccharomyces cerevisiae* showed a positive association with this dietary index (Figure S10).

Other dietary components, including cereals, fruits, milk products, carbohydrates, and energy formed a different cluster that, in contrast to the previous one, is richer in simple sugars such as fructose or lactose. This cluster was mainly positively associated with *Candida dubliniensis, Mitsuokella jalaludinii, Howardella ureilytica*, and a member of Prevotellaceae family (Figure 6). *Collinsella aerofaciens* was also correlated to this cluster but with lower association index (Figure S10, Table S7). Interestingly, *Candida dubliniensis, Mitsuokella jalaludinii*, and Prevotellaceae family presented a remarkably similar pattern of association (Figure S10).

Finally, fats and oils and monounsaturated fatty acid clustered together and negatively correlated with *Malassezia restricta, Subdoligranulum, Lachnoclostridium*, and *Escherichia/Shigella*. However, polyunsaturated fatty acids showed an opposite pattern, directly correlating with the above fungal and bacterial taxa (Figure 6, Figure S10, Table S7). In addition, this lipid cluster was positively associated with *Candida sake, Candida zeylanoides, Pichia kluyveri, Wickerhamomyces onychis*, and the bacterial genus *Faecalibacterium* (Figure 6, Figure S10, Table S7).

## Discussion

In this work, we characterized the fecal mycobiome of PWH and explored its interplay with the bacteriome, diet, and the immune system. To the best of our knowledge, this is the first characterization of the intestinal mycobiome in PWH. We found multiple links between fungal components and markers of innate and adaptive immunity, underscoring the possible influence of the mycobiome on chronic inflammation. Finally, our findings suggested the role of diet in determining the mycobiome composition.

PWH mycobiomes were dominated by a few taxa, as indicated by the low Shannon diversity index and Chao1 estimator. The mycobiota core was defined by yeasts, such as *Saccharomyces, Candida, Debaryomyces, Malassezia*, and *Pichia*. Moreover, alpha diversity was lower for the mycobiome than for the bacteriome, in both PWH and controls. This finding disagrees with a previous report evaluating the mycobiome in PWH and controls, where a major richness was observed in PWH, which could be explained by the fact that authors evaluated a different anatomical site, the palatine tonsil, and by technical issues, such as the use of ITS1 primers, while we amplified the ITS2 region.^18^ The mycobiome field is still challenging due to methodological differences in extraction methods or amplifiable regions, and the poor annotation and the high number of misspellings in fungal databases.

PWH showed an altered mycobiome, in keep with that defined in other inflammatory diseases, such as Crohn’s disease, *Clostridium difficile* infection, or type 2 diabetes.^28–32^ All these diseases are characterized by an enrichment for *Candida* genus and specifically *C. albicans*. In our study, PWH showed enrichment for *C. albicans, C. parapsilosis*, and *D. hansenii*. In contrast, *Malassezia restricta* was depleted in PWH, while in Crohn’s disease this yeast dominates the mycobiome and correlates with disease severity.^33^ *Pichia kluyveri* was also depleted in PWH, in accordance with Mukherjee et al.^14^ that described an enrichment of *Candida* and a depletion of *Pichia* in the oral cavity of PWH. Interestingly, *Penicillium*, which was also depleted in PWH mycobiome, has been described as a xenobiotic degrader resulting in anti-inflammatory metabolites.^34^

Unlike the findings by Fukui et al. in the tonsillar mycobiome,^18^ we observed a different pattern of associations between the mycobiome and bacteriome in PWH and healthy controls. In PWH, bacteria from Ruminococcaceae (*Subdoligranulum* and *Faecalibacterium*) and Lachnospiraceae families directly correlated with *Candida* species. Both *Faecalibacterium* and *Candida*, have been related to a plant-based diet.^35^ However, in healthy controls, *Faecalibacterium prausnitzii* inversely correlated with different fungal species, such as *Malassezia restricta, Rhodotorula mucilaginosa*, and *Meyerozyma guilliermondii*. Interestingly, *Saccharomyces cerevisiae*, the most prevalent yeast in both groups, was not present in the association core. Sokol et al.^28^ had previously revealed different association patterns between the mycobiota and the bacteriome in intestinal bowel disease, where members of Malasseziales inversely correlated with butyrate-producing bacteria and directly with *S. cerevisiae*. Further studies could elucidate the biological significance of the inter-kingdom interactions on the host physiology in health and disease.

The knowledge of the interplay between commensal mycobiome and the immune system is very scarce and the few studies that exist focused on the immune response to a fungal infection. PWH under ART exhibit chronic immune activation and an altered bacterial microbiota, which have been associated with this inflammatory status.^1–5^ The main butyrate-producer bacteria, such as *Faecalibacterium, Lachnospira*, and other genera in the Lachnospiraceae and Ruminococcaceae families could be involved in the immune response, affecting IL-17, IL-22 and IFABP expression. As indicated in previous studies,^11,12^ we found that the fungal cell wall component from yeasts, such as *Saccharomyces* and *Candida* could induce the adaptive immunity by increasing the production of sTNF-R2, IL-17 or IL-22. In our study, the clearest correlations between the mycobiome and immune markers were found between species of *Candida* (*C. sake, C. albicans, C. zeylanoides, C. glabrata, C. tropicalis*) and total lymphocyte counts, CD8 + T cell counts and senescent CD8 + T cells, suggesting a role of *Candida spp*. in immune activation.

The high inter-individual variability of the fecal mycobiome has been related to the transient nature of some fungal taxa depending on factors, such as diet and lifestyle.^8,35^ However, to our knowledge, the impact of diet on the intestinal fungal communities remained unexplored. Previous works in different settings had suggested that fungi such as *Saccharomyces, Ustilago*, or *Agaricus* could be associated with the recent consumption of certain foods.^35^ Here, we identified an inter-kingdom consortium consisting of bacteria, such as *Prevotella* and *Howardella* and yeasts, such as *Candida* and *Pichia* that would be capable to degrade vegetable fiber. *Faecalibacterium* and *Subdoligranum* would be part of this consortium as short-chain fatty acid-producing bacteria from the simple sugars yielded from fiber degradation and *Candida* and *Hanseniaspora* would also ferment these monosaccharides. By-products, including H2 and CO2, could be consumed by *Collinsella* which would be included in the consortium since these actinobacteria correlated with cereals, fruits, milk, carbohydrate, and energy cluster. In addition, our findings suggested that *C. sake, C. zeylanoides, Pichia kluyveri, Wickerhamomyces onychis*, and *Faecalibacterium* together would play a role in the metabolism of monosaturated fatty acids and lipids. However, other bacterial and fungal species were associated with polyunsaturated fatty acids.

The major strength of our study is its novelty since the inter-kingdom relations in PWH related to diet and their potential impact on systemic immunity had not been studied before. The main limitations are the small sample size of the groups and the impossibility of establishing causal relationships between variables due to the cross-sectional design. In addition, sexual orientation is a significant driver of the differences in bacterial communities firstly attributed to HIV.^36,37^ Due to the limited sample size, we could not address the potential confounding factor of sexual orientation on the mycobiome composition. Whether the sexual orientation affects the fungal communities composition or not remains unknown. Although, our research is focused on microbiome changes during treated infection as a potential driver of persistent immune defects, the inclusion of an ART naïve group could provide some interesting information. Moreover, the high-throughput sequencing based on DNA amplicons does not allow distinguishing the origin of the fungus.

In conclusion, despite the great difference in abundance and diversity between the bacterial and fungal communities of the gut, we established relevant interactions between both kingdoms, found an effect of HIV, and connections with diet and systemic inflammation. Future studies on this interplay will allow for a better understanding of the chronic immune activation in PWH. Moreover, the findings support the crucial role of the mycobiome in nutrient metabolism. Further investigation on gut inter-kingdom consortia and diet is still needed to exploit the mutual interactions to improve human health.

## Patients and methods

### Subjects and samples

Participants were on antiretroviral therapy with plasma HIV RNA < 37 copies/mL during at least 48 weeks and presented a chronic immune activation (CD4/CD8 ratio < 1). Only one patient received antifungal treatment 165 weeks before the study (Table 1). The study samples were collected during 2017. The study was conducted according to the guidelines of the Declaration of Helsinki and approved by the Ethics Committee of University Hospital Clínico San Carlos (approval number: 11/284; Clinical Trials Registry Identification Number Identifier: NCT01838915) and University Hospital Ramón y Cajal (approval number: 165/16; Clinical Trials Registry Identification Number Identifier: NCT03008941). All participants signed an informed consent before the initiation of study procedures.

### Determination of clinical variables

The plasma levels of inflammatory biomarkers were determined from the cryopreserved plasma by immunoassay in triplicate by following the manufacturer instructions of the kits: Tumor soluble necrosis factor sTNF-receptor 2 (sTNF-R2) (DRT200, R&D Systems, Bio-Techne Corporation, Minneapolis, MN, USA), C-reactive protein (CRP) (DCRP00, Quantikine ELISA kit, R & D Systems, Minneapolis, MN, USA), sCD14 (AbClonal, Wuhan, China), sCD163 (AbClonal, Wuhan, China), FABP2/IFABP (Boster Biological Technology, Wuhan, China), D-dimers (Ray Biotech, Norcross, GA, USA), LTA (Abbexa, Cambridge, UK), LBP (Boster Biological Technology, Wuhan, China), IP-10 (DIP100, R&D Systems, Bio-Techne Corporation, Minneapolis, MN, USA), IL-17 (Sigma Aldrich, Misuri, US), and IL-22 (Novateinbio, Massachussetts, USA).

T-cell immunophenotyping from thawed PBMCs was performed with the following antibody combination: CD3-VioBlue, CD4-Fluorescein isothiocyanate (FITC), CD8-VioGreen, CD28-Phycoerythrin (PE), CD38-APC and HLA-DR-APC-Vio770, and PD-1 (PD-1-PE-Vio770). Antibodies were purchased from Myltenyi Biotec (Bergisch Gladbach, Germany), and isotype controls were carried out. Cells were analyzed using a Gallios flow cytometer (Beckman-Coulter, CA, USA).

### Food and nutrient intake data

A three-day dietary record including a Sunday (from Sunday to Tuesday) was used to determine all foods and beverages consumed by adults during that time period. Participants were instructed to record all the food, beverages, and supplements consumed during the pre-established period. Participants were informed in a clear and precise way about how they should record all the information in detail, including the methods of food preparation and the ingredients in dishes and recipes. The importance of not forgetting to record the food consumed between meals (snacks, sweets, etc.), as well as the consumption of bread or sweeteners was stressed. To minimize mistakes after data collection, all interviews were reviewed by the study dietitians to assess unrealistic portion sizes, inadequate details, and typing errors. This prospective dietary method has been previously validated and is a method widely accepted to collect dietary information.^38–40^ Energy and nutrients intake from the food and beverages consumed were calculated using the DIAL software (v3.0.0.12), through the data from the Spanish Food Composition Tables.^41^ In addition, the percentages of energy to total energy intake contributed by macronutrients, saturated fat, polyunsaturated fat, and monounsaturated fat were calculated. Furthermore, participants reported the frequency of consumption through the Food Frequency Questionnaire in the last year. The data obtained served to categorize individuals according to their usual food consumption, in addition to contrasting the information obtained with the other method of collecting dietary data used.^42^ MED-DQI was calculated for each patient, taking into account the following components: % of saturated fatty acids in relation to total energy, cholesterol (mg), grams of meat, ml of olive oil, grams of fish, and grams of fruit and vegetables. Each nutrient or food group was assigned three scores (0, 1, and 2) based on the recommended guidelines. MED-DQI scores between 1 and 4 are considered good, between 5 and 7 medium good, between 8 and 10 medium poor, and 11–14 poor.^43^

### Total DNA extraction and sequencing

Fecal samples were centrifuged at 2000 rpm at 4°C for 2 min to remove fecal debris. The cellular suspensions were treated with a lytic solution (lysozyme (0.1ug/ul) and zymolyase (7mU/ul)) at 37°C for 1 h with gently shaking. After proteinase K digestion, we performed three freeze/boil cycles (using dry ice and a heating block) to increase the lysis efficiency. Then, the DNA extraction was performed in the robotic workstation MagNA Pure LC Instrument (Roche) using the MagNA Pure LC DNA isolation kit III (Bacteria, Fungi) (Roche).

To analyze the mycobiome composition, we amplified the Internal Transcribed Spacer 2 (ITS2) region of the rRNA operon using the primers ITS3-F and ITS4-R described in Nash et al.^8^ PCR conditions were: initial denaturation step at 95°C for 3 min, 30 amplification cycles of 95°C for 30 s,58°C for 30 s, and 72°C for 30 s, followed by an extension step of 72°C for 5 min. The sequencing libraries were constructed following Illumina instructions and sequenced using the Kit v3 (2x230 cycles) in a MiSeq platform (Illumina) at the FISABIO Sequencing and Bioinformatics Service, Valencia, Spain. All the fungal sequences have been deposited in the EBI database under the number PRJEB46343.

### Sequence analyses

To analyze the ITS amplicons, we adapted the ITS version of the DADA2 workflow.^44^ First, we removed the reverse complement form of ITS3-F-ITS4-R primers using cutadapt tool (v1.18).^45^ Next, we applied Prinseq (v0.20.4) for trimming the reads with bases with quality lower than 30 and for discarding the reads shorter than 100 nucleotides.^46^ The following steps were performed with R (v3.6.0) by means of the corresponding functions of the DADA2 library (v1.8.0).^47^ Dereplication was carried out to combine all identical reads into unique sequences. Taking the dereplicated reads and the error estimations, sequence variants were inferred. The forward and reverse pairs were merged together to obtain the single denoised variants. The chimera sequences were identified and discarded, resulting in the final amplicon sequence variants (ASVs). The UNITE ITS database (v8.0) was set as the reference for assigning taxonomy to each fungal ASV.^48^ The remaining not classified ASVs, were mapped with the Blastn tool (v2.9.0+) against three different NCBI-Refseq databases of complete genomes: Fungi (January 2018), plants (March 2019), and bacteria (May 2019).^49,50^ The best alignments were manually revised and the taxonomy assigned to the previously not classified ASVs.

To analyze the bacterial community of fecal samples (bacteriome), we use the raw 16S rRNA gene reads obtained in previous studies and deposited in the EBI database under the number PRJEB25569 for control samples and under PRJEB36786 for PWH samples.^10,51^ The 16S rRNA gene reads were processed using the DADA2 pipeline in the R package. Also, this pipeline was used to create the amplicon sequence variants. The taxonomic information of the ASVs was obtained by BLAST comparison against the SILVA reference database (v.132).^52^

The Shannon diversity index and Chao1 estimator at ASV level were obtained with vegan library from R package. Mycobiome composition was studied at genus and species level and the barplots represented those taxa that were present at least in 25% of the samples.

As the mycobiome ASV table contained many zeros and to avoid numerical irregularities, a smoothing step was separately performed in each sample groups. Also, we applied an arcsine square root-transformation to better approximate normality. The bacterial ASVs were normalized by total-sum scaling. To assess the beta-diversity, the Bray-Curtis dissimilarity index, PCoA, clustering, and heatmaps were generated with in-house R scripts (v3.1).

Linear Discriminant Analysis Effect Size algorithm was applied to identify taxonomical biomarkers in bacteriome.^53^ Default parameters were used for significance (p-value < 0.05) and linear discriminant analysis threshold (<2.0). In addition, we performed pairwise Wilcoxon rank-sum tests for statistical significance between groups, applying an adjustment for multiple comparisons using the Benjamini–Hochberg correction. Adonis test, a multivariate analysis of variance based on dissimilarity, was applied using R package (adonis function) to statistically assess the effect of external factors on microbial composition.

### Discriminant analysis

We applied sparse Partial Least Square-discriminant analysis (sPLS-DA) using mixOmics package in R to select fungal ASVs with a high discriminative capacity to classify the samples in one of the groups, PWH or HIV-.^54^ First, we chose, with the “perf” function (n repeats = 50), the number of components in the model, based on the estimation of the classification error rate using cross-validation, being 2 the number of components chosen in our study. Then, we estimated the number of selected variables per component in the model with the function tune.splsda (n repeats = 50). Thus, our final sPLS-DA model included two components and 16 and 4 variables on component 1 and component 2, respectively. Finally, the sPLS-DA model was evaluated using the “perf” function (5-fold cross-validation and 50 repeats) and ROC curves (Figure S11). The accuracy has been calculated as 1 – classification error rate.

### Association analysis sPLS

To assess potential interactions between compositional data and clinical or diet features, we use mixOmics package in R.^54^ We compute pairwise associations between features based on sparse Partial Least Square (sPLS) models that are very flexible models allowing large number of quantitative variables to be associated with a response that could be both univariate and multivariate, both quantitative and qualitative. We used sPLS with ncomp = 3 and 50 or 20 variables per component (KeepX) and we applied a canonical mode since this method models bi-directional (no causal) relationships between two data sets.^55^ The association index is obtained as the inner product of the vectors of each variable derived from the coefficients obtained in principal component analysis. To represent the associations as networks and heatmaps, a similarity matrix is calculated from the outputs of PLS and the function ‘ggraph’ in the package R was used to visualize the graphics.^56^

## Supplementary Material

Supplemental Material

## Data Availability

The datasets used and/or analyzed during the current study are available from the corresponding author on reasonable request. The fungal sequences generated and analyzed during the current study are available in the EBI database under the number PRJEB46343 [https://www.ebi.ac.uk/ena/browser/view/PRJEB46343].
