# Supplementary material for: Interactions among the mycobiome, bacteriome, inflammation, and diet in people living with HIV
Source: Gut Microbes. 2022 Jun 23;14(1):2089002. doi: 10.1080/19490976.2022.2089002 (PMC9235884; doi:10.1080/19490976.2022.2089002)
Supplement: Supplemental Material [file KGMI_A_2089002_SM7564.zip › SupplementaryMaterials/FigureS6.pdf]

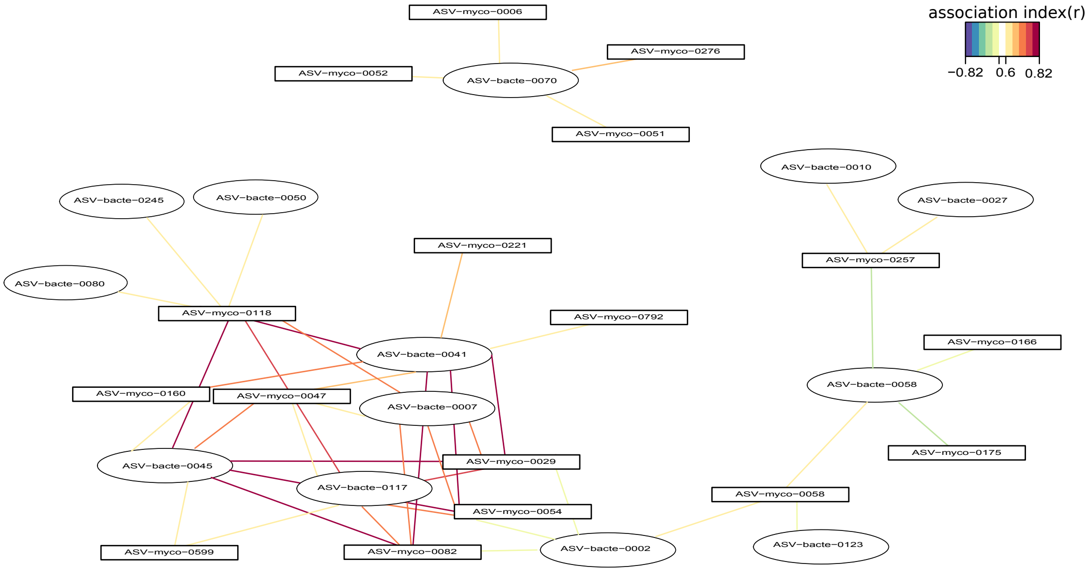

ASV-bacte-0002	*Faecalibacterium prausnitzii*	ASV-myco-0006	*Dipodascus australiensis*
ASV-bacte-0007	*Subdoligranulum*	ASV-myco-0029*	*Rhodotorula mucilaginosa*
ASV-bacte-0010	*Faecalibacterium CM04-06*	ASV-myco-0047*	*Malassezia restricta*
ASV-bacte-0027	*Parabacteroides merdae*	ASV-myco-0051	Dipodascaceae
ASV-bacte-0041	*Bacteroides uniformis*	ASV-myco-0052	Dipodascaceae
ASV-bacte-0045	*Lachnoclostridium*	ASV-myco-0054*	*Meyerozyma guilliermondii*
ASV-bacte-0050	*Bacteroides*	ASV-myco-0058	*Candida sake*
ASV-bacte-0058	*Coprococcus comes*	ASV-myco-0082*	*Malassezia restricta*
ASV-bacte-0070	*Bifidobacterium*	ASV-myco-0118*	*Kwoniella botswanensis*
ASV-bacte-0080	*Lachnoclostridium*	ASV-myco-0160*	*Naganishia friedmannii*
ASV-bacte-0117	*Oscillibacter*	ASV-myco-0166	*Pichia kluyveri*
ASV-bacte-0123	*Blautia obeum*	ASV-myco-0175	*Penicillium glabrum*
ASV-bacte-0245	*Odoribacter splanchnicus*	ASV-myco-0221	*Psathyrella candolleana*
		ASV-myco-0257	*Saccharomyces cerevisiae*
		ASV-myco-0276	*Candida quercitrusa*
		ASV-myco-0599	*Phoma saxea*
		ASV-myco-0792	*Fusarium*
