# Supplementary figures and images for: Interactions among the mycobiome, bacteriome, inflammation, and diet in people living with HIV
Source: Gut Microbes. 2022 Jun 23;14(1):2089002. doi: 10.1080/19490976.2022.2089002 (PMC9235884; doi:10.1080/19490976.2022.2089002)

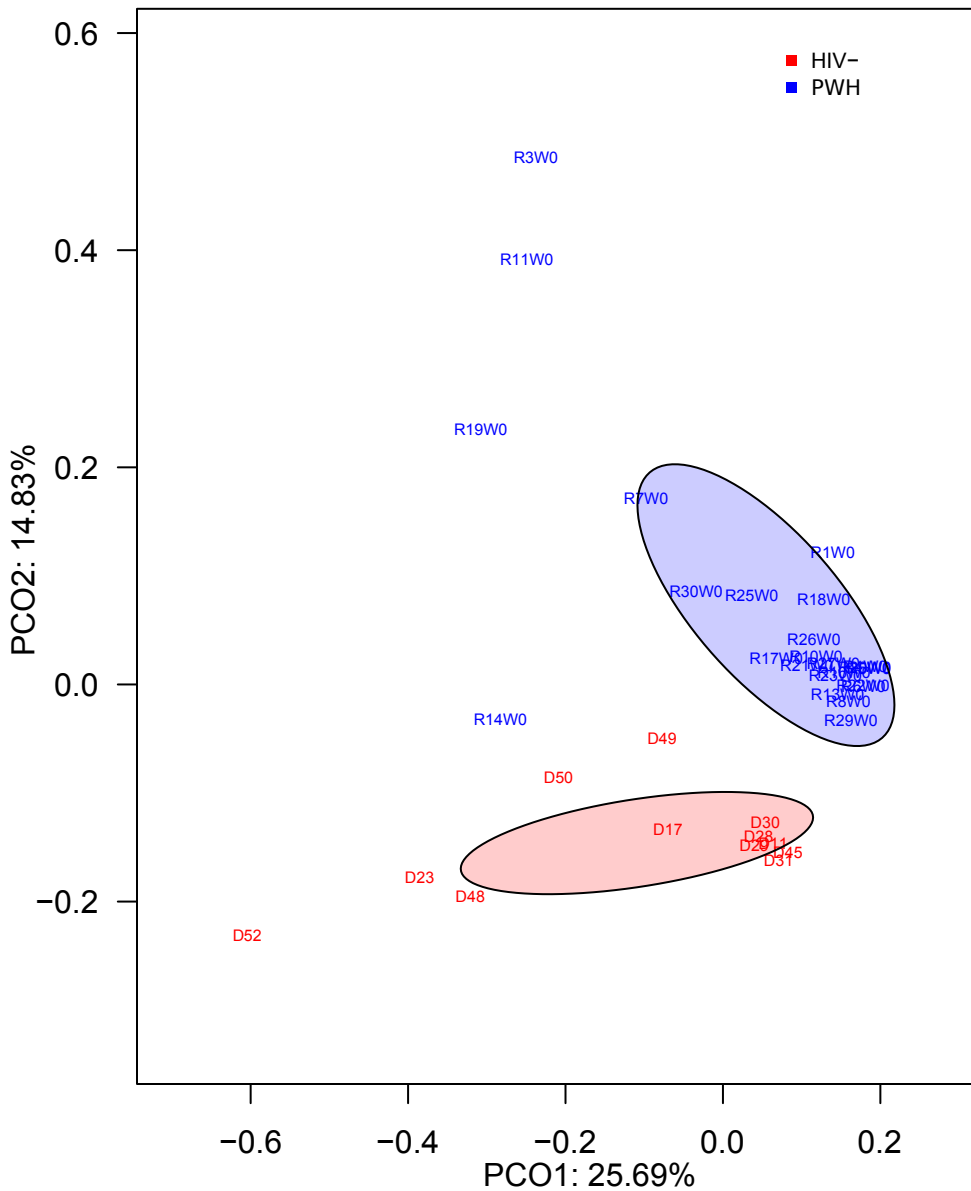

Supplement: Supplemental Material [file KGMI_A_2089002_SM7564.zip › SupplementaryMaterials/FigureS1.pdf]

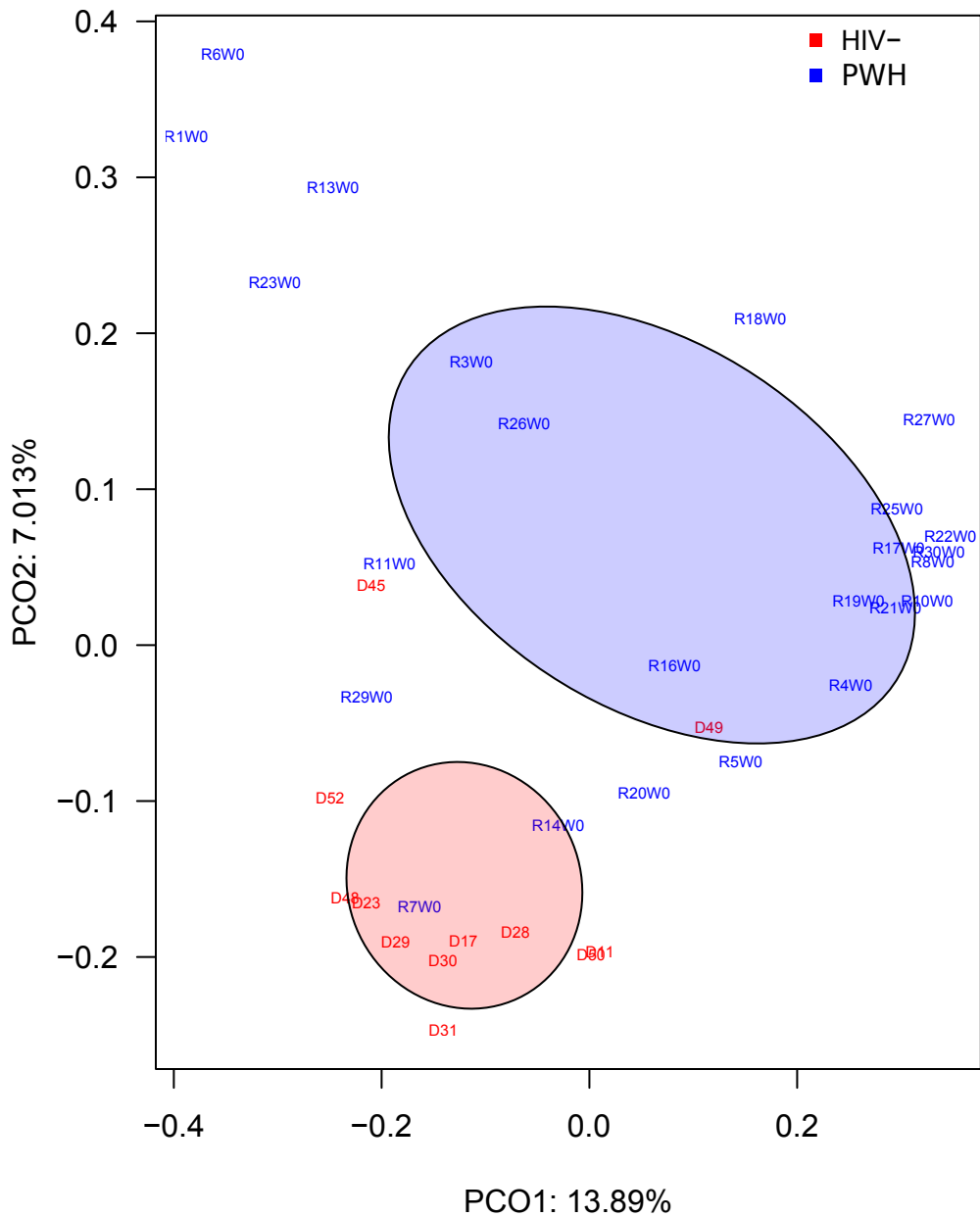

Supplement: Supplemental Material [file KGMI_A_2089002_SM7564.zip › SupplementaryMaterials/FigureS3.pdf]

association index (r)

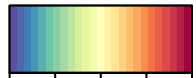

-0.83 0 0.83

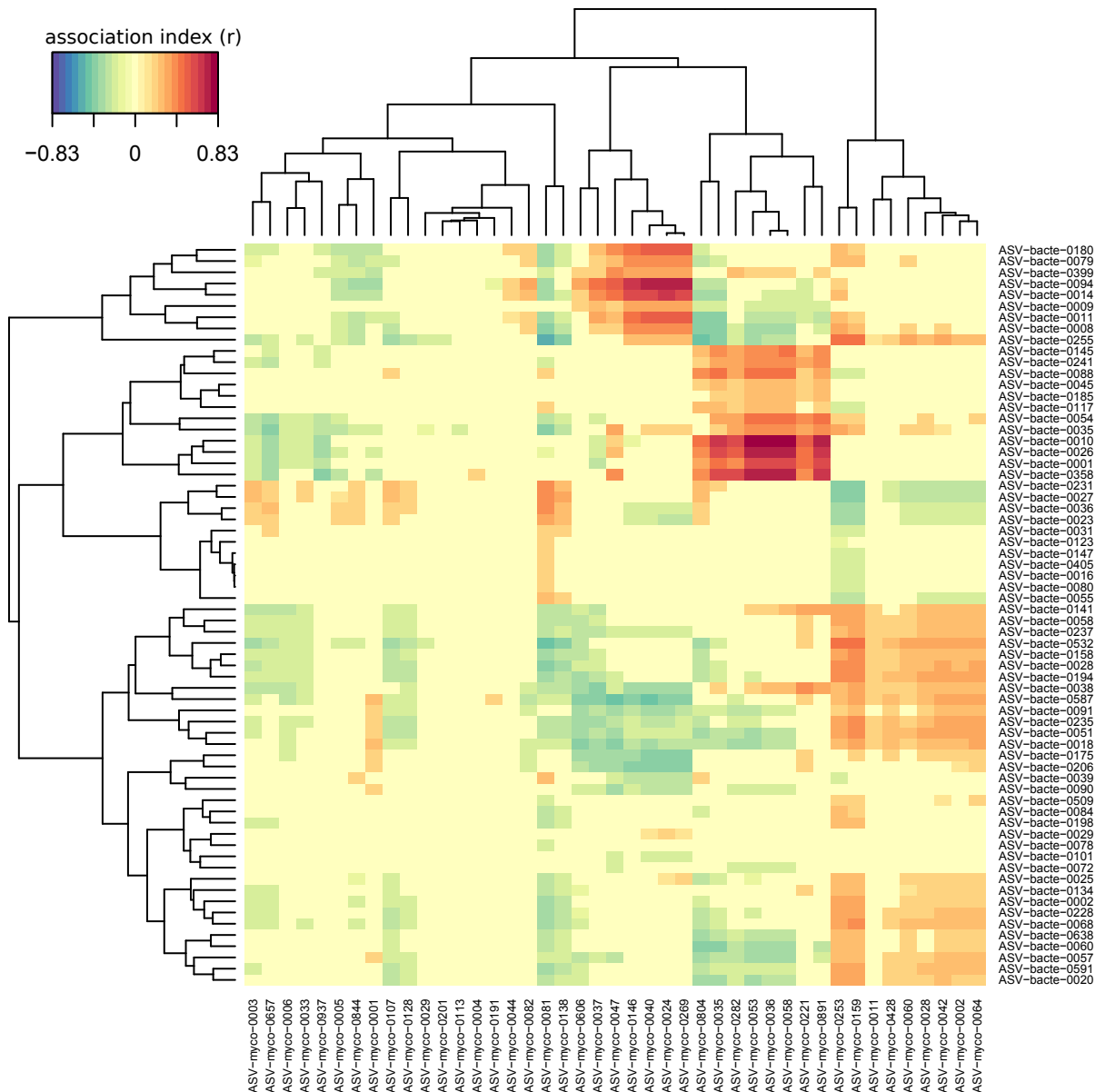

Supplement: Supplemental Material [file KGMI_A_2089002_SM7564.zip › SupplementaryMaterials/FigureS5.pdf]

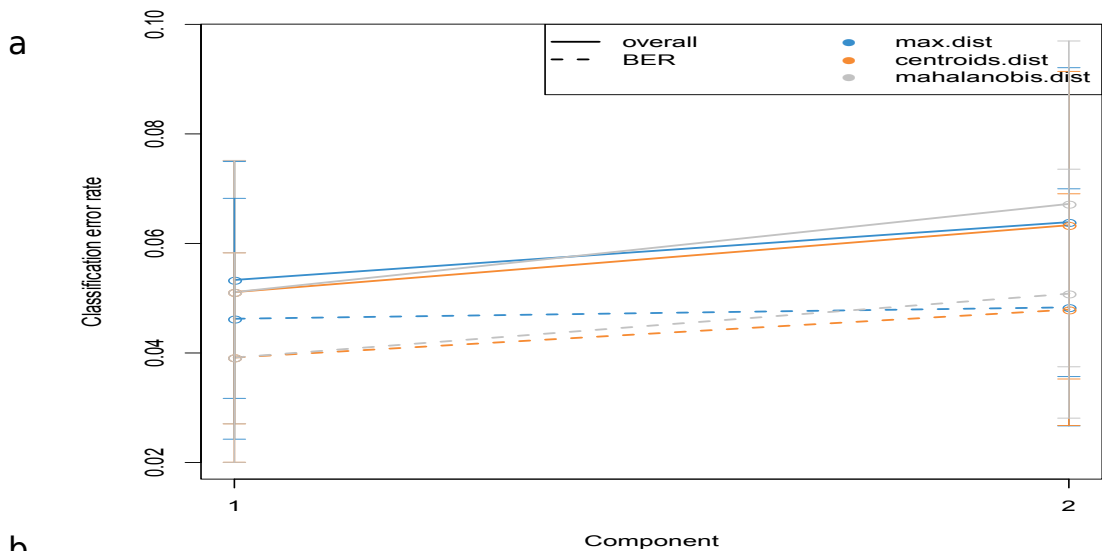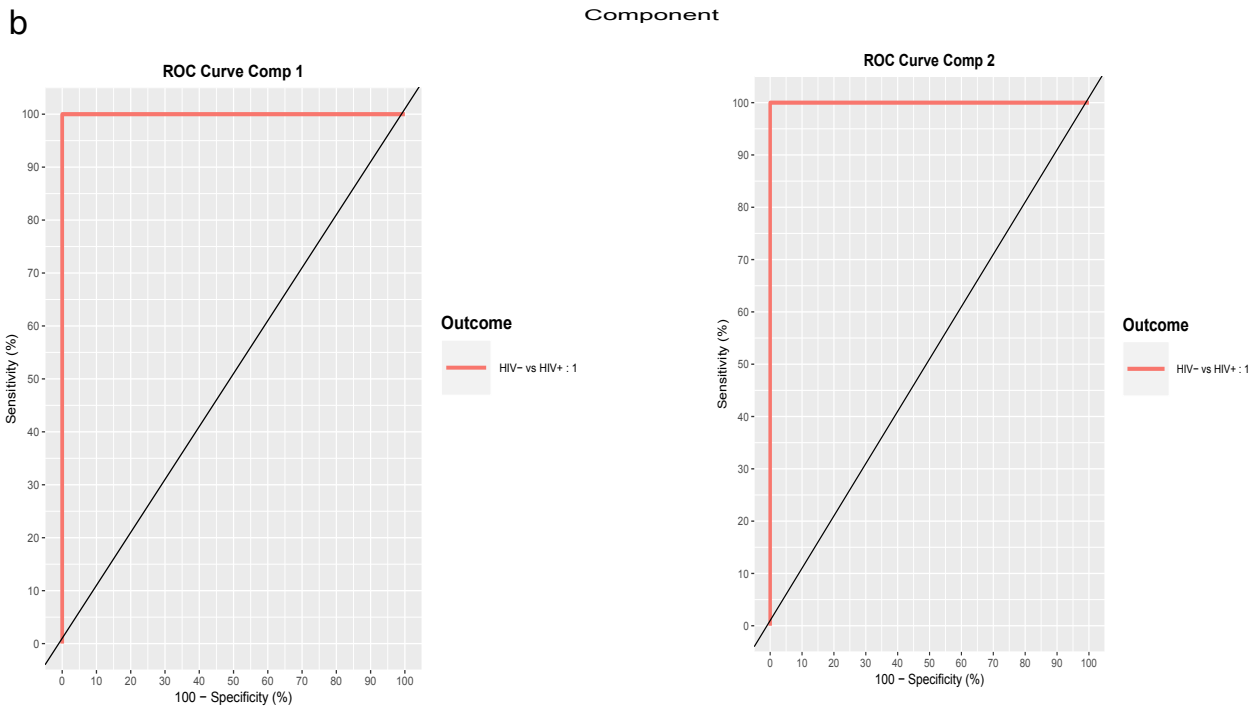

Supplement: Supplemental Material [file KGMI_A_2089002_SM7564.zip › SupplementaryMaterials/FigureS11.pdf]

association index (r)

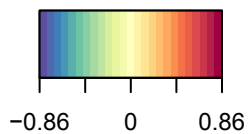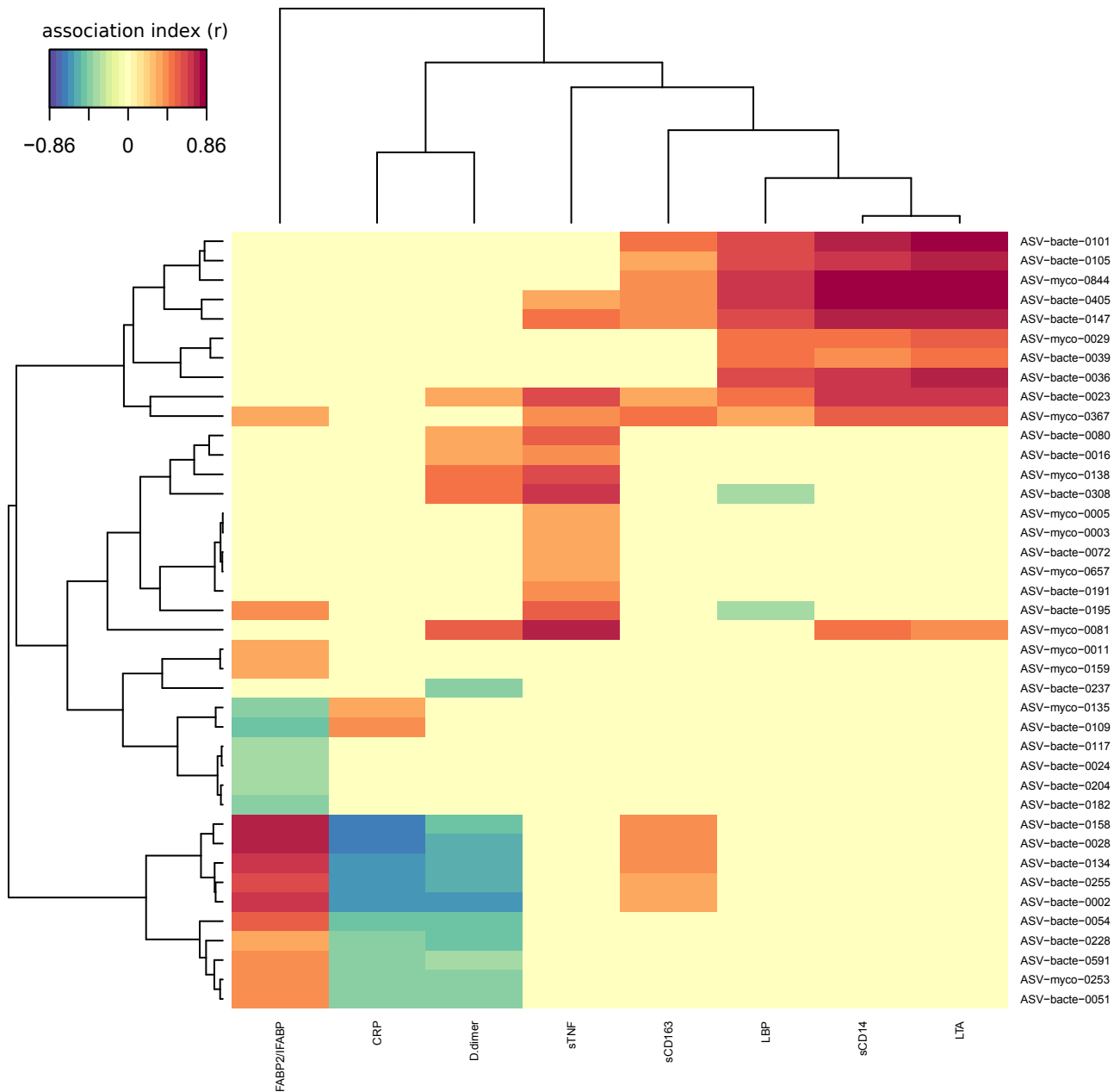

Supplement: Supplemental Material [file KGMI_A_2089002_SM7564.zip › SupplementaryMaterials/FigureS7.pdf]

association index (r)

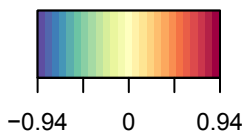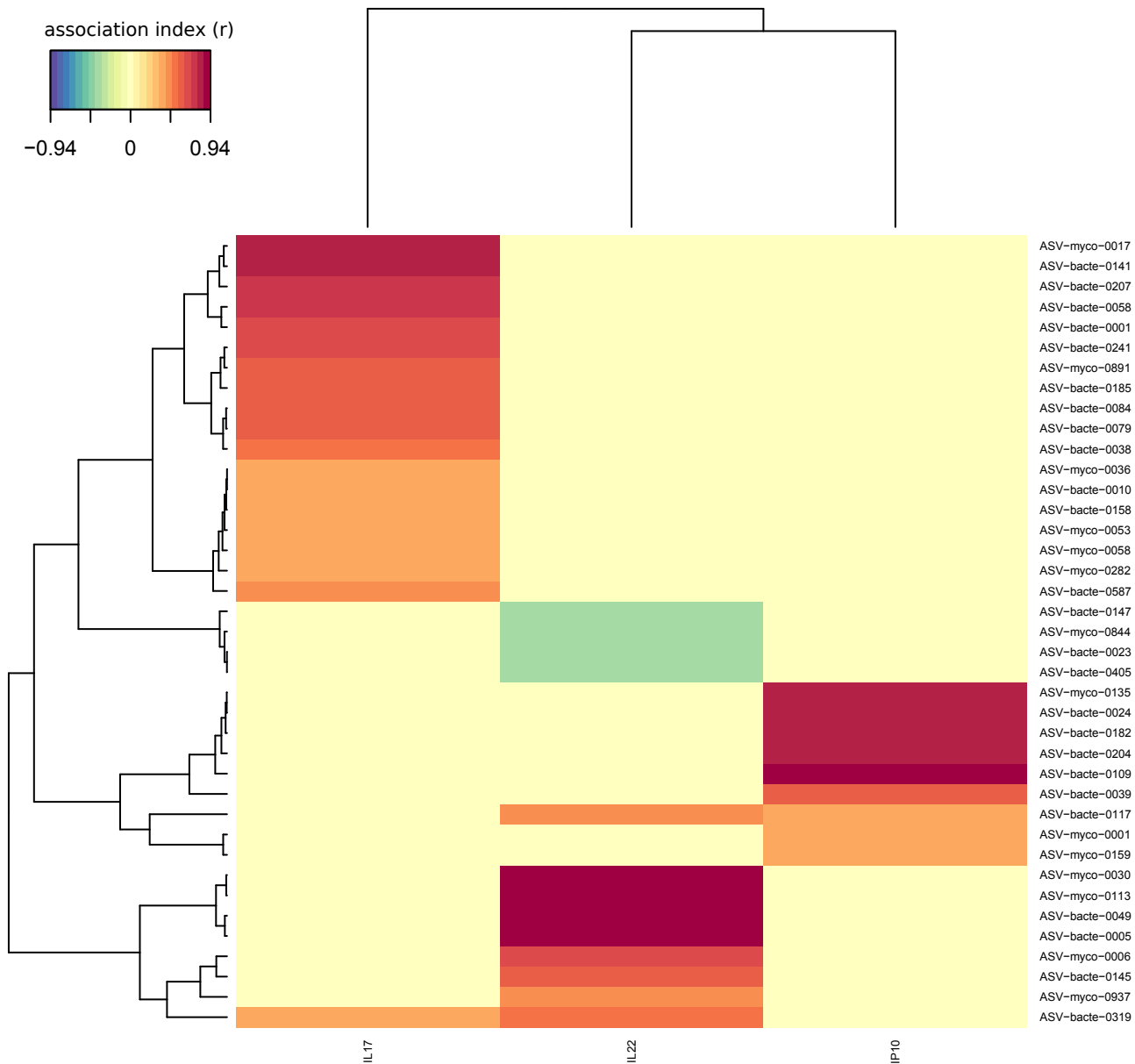

Supplement: Supplemental Material [file KGMI_A_2089002_SM7564.zip › SupplementaryMaterials/FigureS8.pdf]

association index ( $r$ )

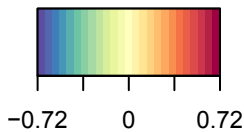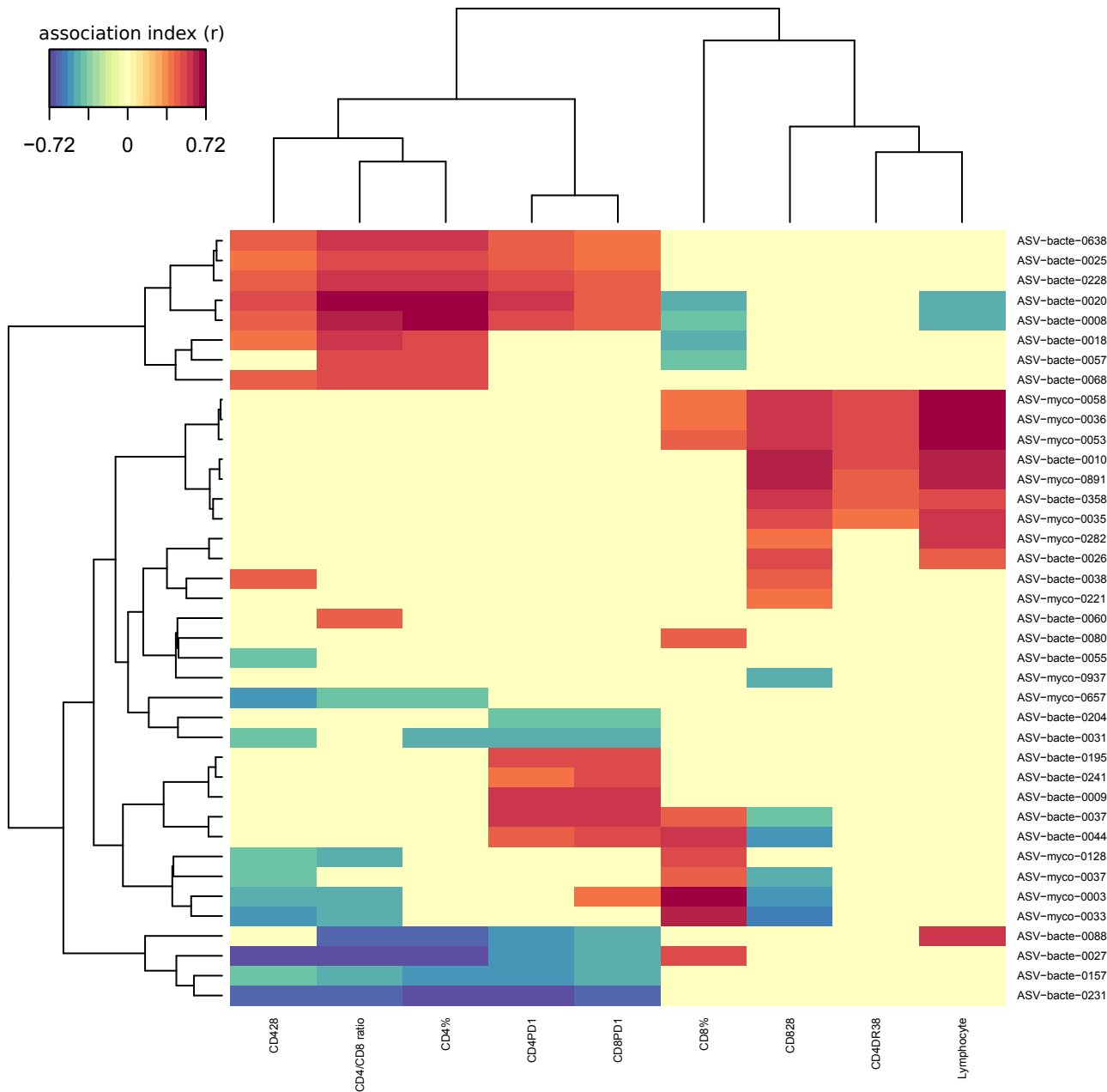

Supplement: Supplemental Material [file KGMI_A_2089002_SM7564.zip › SupplementaryMaterials/FigureS9.pdf]

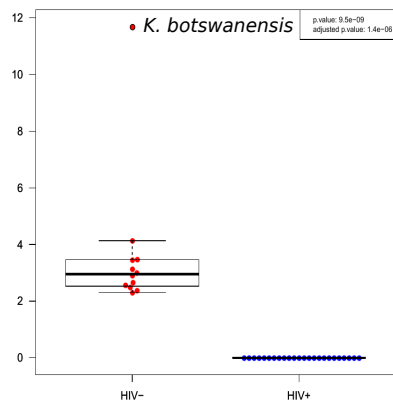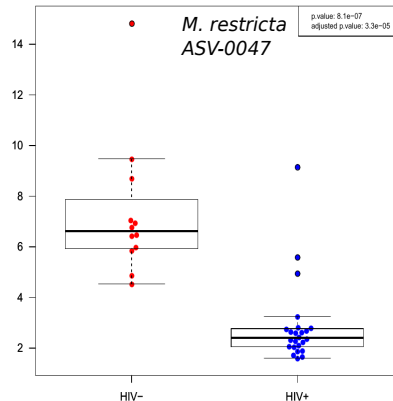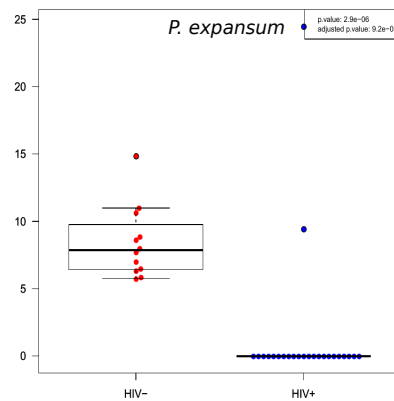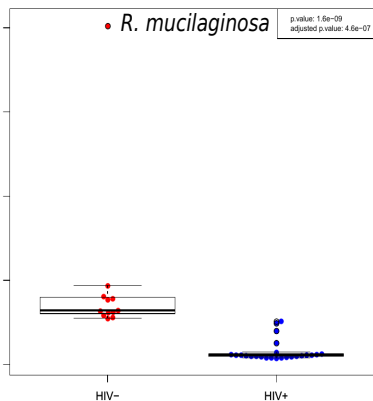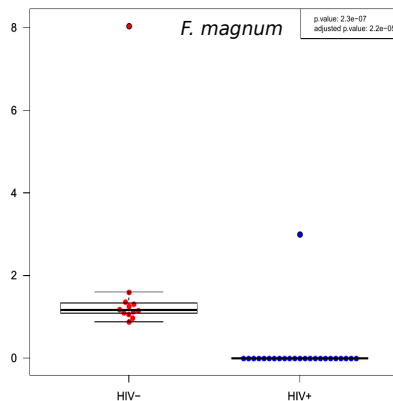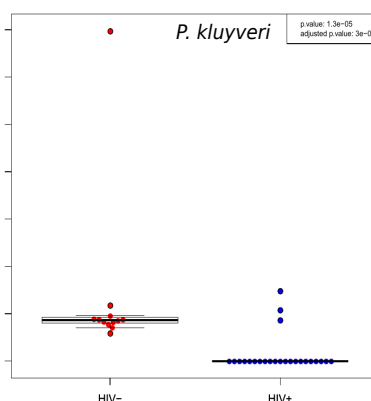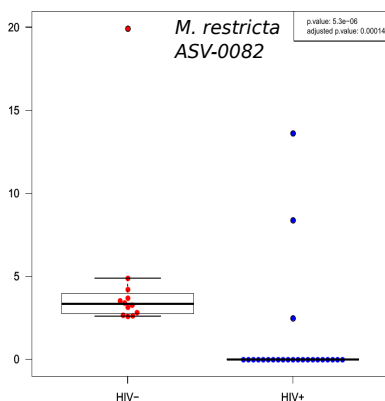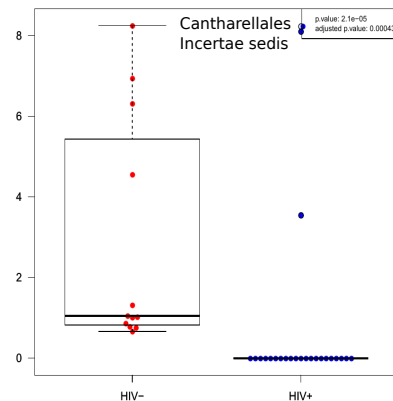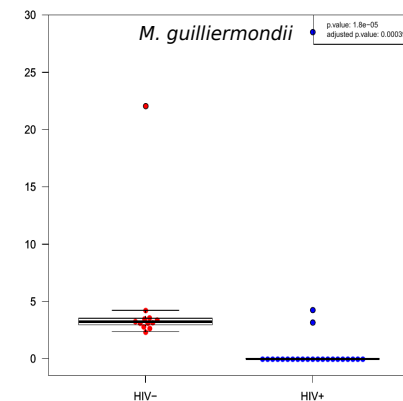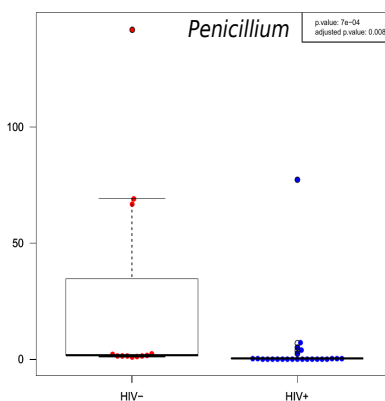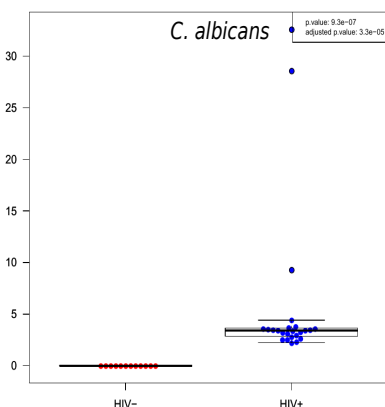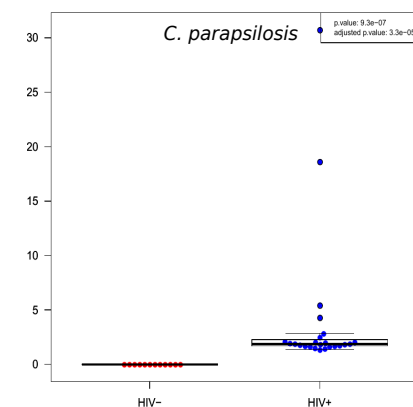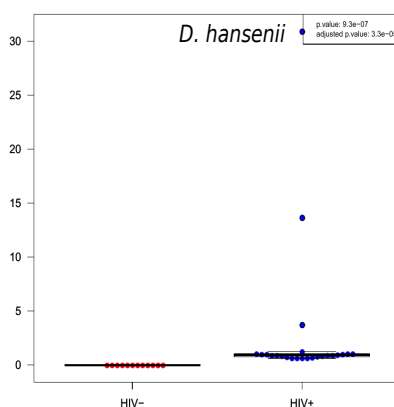

Supplement: Supplemental Material [file KGMI_A_2089002_SM7564.zip › SupplementaryMaterials/FigureS2.pdf]

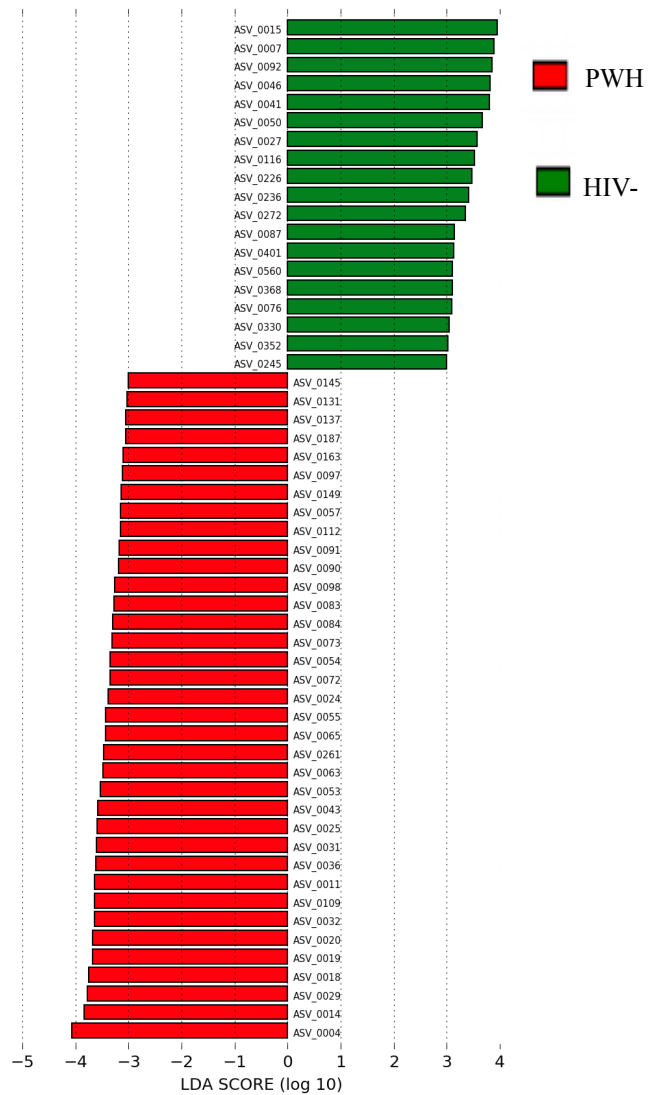

Supplement: Supplemental Material [file KGMI_A_2089002_SM7564.zip › SupplementaryMaterials/FigureS4.pdf]

association index (r)

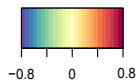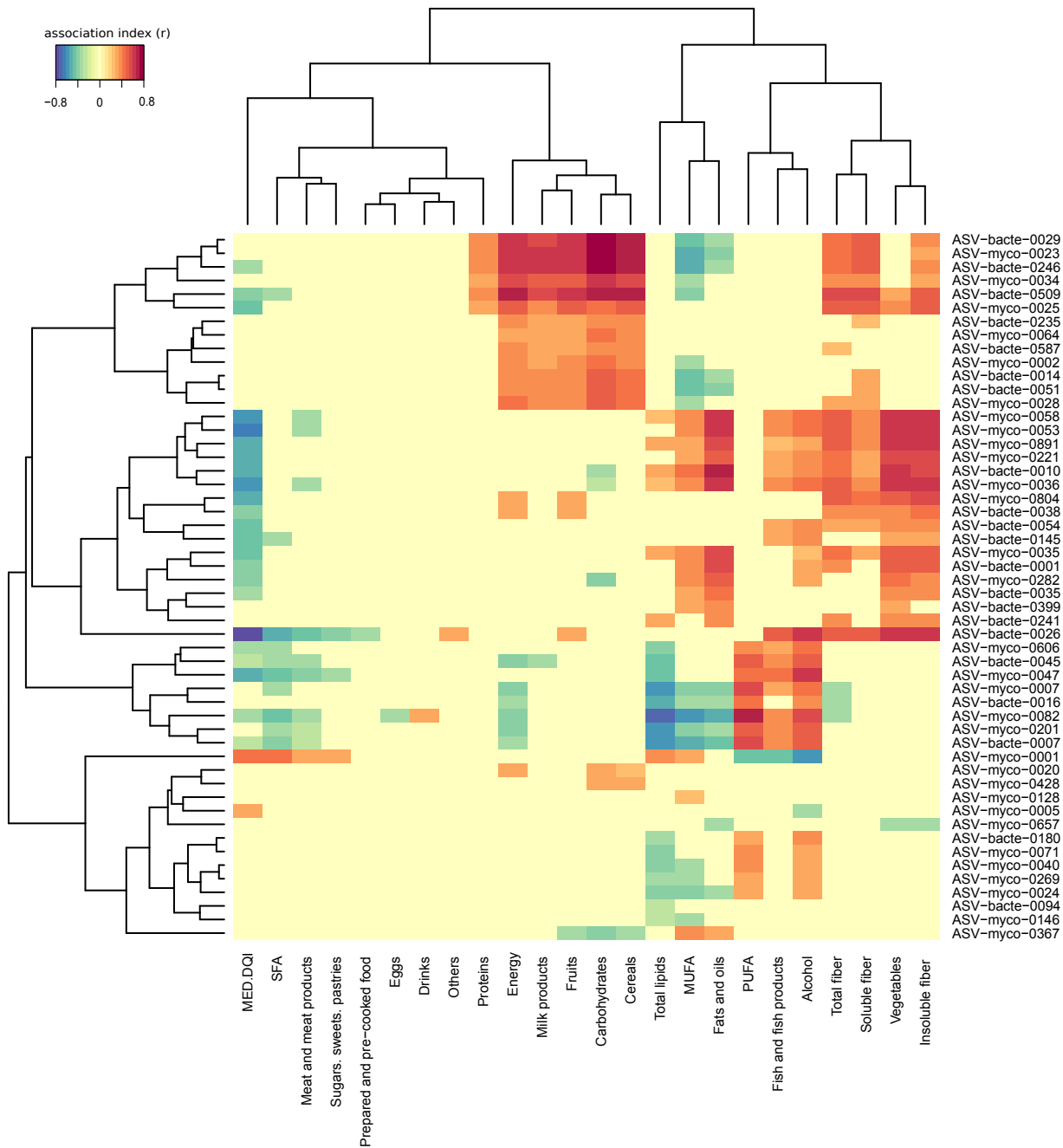

Supplement: Supplemental Material [file KGMI_A_2089002_SM7564.zip › SupplementaryMaterials/FigureS10.pdf]
