# Supplementary material for: Interactions among the mycobiome, bacteriome, inflammation, and diet in people living with HIV
Source: Gut Microbes. 2022 Jun 23;14(1):2089002. doi: 10.1080/19490976.2022.2089002 (PMC9235884; doi:10.1080/19490976.2022.2089002)
Supplement: Supplemental Material [file KGMI_A_2089002_SM7564.zip › SupplementaryMaterials/SupplementaryFigures.doc]

**Supplementary Materials:**

**Title:** Interactions among mycobiome, bacteriome, inflammation and diet in people living with HIV

**Authors:** María José Gosalbes, Nuria Jimenéz-Hernandéz, Elena Moreno, Alejandro Artacho, Xavier Pons, Sonia Ruíz-Pérez, Beatriz Navia, Vicente Estrada, Mónica Manzano Alba Talavera-Rodriguez, Nadia Madrid, Alejandro Vallejo, Laura Luna, José A. Pérez-Molina, Santiago Moreno, Sergio Serrano-Villar

**The following supporting information is available for this article:**

**Figure S1:** sPLS-DA plot on the two first components. The first component discriminated both groups, PWH and HIV-.

**Figure S2:** Boxplots of the taxa detected by sPLS-DA that presented significantly different abundance between both groups.

**Figure S3:** Beta diversity of the two bacteriomes. PCoA of the bacterial communities from HIV- and PWH groups based upon Bray-Curtis dissimilarities. Adonis test: p-value 0.0016.

**Figure S4:** LefSe analysis of bacterial ASVs between HIV- group (green) and PWH subjects (red). LDA scores (log10) for the most prevalent ASVs in the HIV- group are represented on a positive scale, belonging the ASV_0401, ASV_0368, ASV_0050, ASV_0076, ASV_0015, ASV_0041 to *Bacteroides* genus. Negative LDA scores indicate enriched ASVs in the PWH group, belonging ASV_0083, ASV_0131, ASV_0072, ASV_0149, ASV_0011, ASV_0020, ASV_0025, ASV_0043, ASV_0057, ASV_0090, ASV_0098, ASV_0137, ASV_0163 to *Prevotella* genus .

**Figure S5:** Heatmap and clustering of the associations between mycobiome and bacteriome in PWH group.

**Figure S6:** Association network between mycobiome and bacteriome by applying sPLS analysis in HIV- group. Association index >0.6.

**Figure S7:** Heatmap and clustering of the associations between microbiome and inflammatory markers in the PWH group.

**Figure S8:** Heatmap and clustering of the associations between microbiome and cytokines in the PWH group.

**Figure S9:** Heatmap and clustering of the associations between microbiome and T cells in the PWH group.

**Figure S10:** Heatmap and clustering of the associations between microbiome and foods and nutrient intake in the PWH group. ASV-myco-0001 and ASV-myco-0005 correspond to *Saccharomyces cerevisiae*; ASV-myco-0023 to *Candida dubliniensis*; ASV-bacte-0246 to Prevotellaceae family; ASV-bacte-0029 to *Mitsuokella jalaludinii;* ASV-bacte-0051 to *Collinsella aerofaciens* and ASV-myco-0036 to *Candida sake.*

**Figure S11:** Classification performance of the sPLS-DA model. (a) classification error rate. Overall, overall error rate; BER, balanced error rate; max.dist, maximum distance, centroids. dist, centroid distance; mahalanobis.dist, mahalanobis distance, BER with maximum distance indicated a good performance (error rate =0.045). (b) ROC curves for the two first components.

**Supplementary figures**

**Figure S1:** sPLS-DA plot on the two first components. The first component discriminated both groups, PWH and HIV-.

**Figure S2:** Boxplots of the taxa detected by sPLS-DA that presented significantly different abundance between both groups.

**Figure S3:** Beta diversity of the two bacteriomes. PCoA of the bacterial communities from HIV- and PWH groups based upon Bray-Curtis dissimilarities. Adonis test: p-value 0.0016.

**Figure S4:** LefSe analysis of bacterial ASVs between HIV- group (green) and PWH subjects (red). LDA scores (log10) for the most prevalent ASVs in the HIV- group are represented on a positive scale, belonging the ASV_0401, ASV_0368, ASV_0050, ASV_0076, ASV_0015, ASV_0041 to *Bacteroides* genus. Negative LDA scores indicate enriched ASVs in the PWH group, belonging ASV_0083, ASV_0131, ASV_0072, ASV_0149, ASV_0011, ASV_0020, ASV_0025, ASV_0043, ASV_0057, ASV_0090, ASV_0098, ASV_0137, ASV_0163 to *Prevotella* genus .

**Figure S5:** Heatmap and clustering of the associations between mycobiome and bacteriome in PWH group.

**Figure S6:** Association network between mycobiome and bacteriome by applying sPLS analysis in HIV- group. Association index >0.6.

**Figure S7:** Heatmap and clustering of the associations between microbiome and inflammatory markers in the PWH group.

**Figure S8:** Heatmap and clustering of the associations between microbiome and cytokines in the PWH group.

**Figure S9:** Heatmap and clustering of the associations between microbiome and T cells in the PWH group.

**Figure S10:** Heatmap and clustering of the associations between microbiome and foods and nutrient intake in the PWH group. ASV-myco-0001 and ASV-myco-0005 correspond to *Saccharomyces cerevisiae*; ASV-myco-0023 to *Candida dubliniensis*; ASV-bacte-0246 to Prevotellaceae family; ASV-bacte-0029 to *Mitsuokella jalaludinii;* ASV-bacte-0051 to *Collinsella aerofaciens* and ASV-myco-0036 to *Candida sake.*

**Figure S11:** Classification performance of the sPLS-DA model. (a) classification error rate. Overall, overall error rate; BER, balanced error rate; max.dist, maximum distance, centroids. dist, centroid distance; mahalanobis.dist, mahalanobis distance, BER with maximum distance indicated a good performance (error rate =0.045). (b) ROC curves for the two first components.
