# Supplementary material for: Interactions among the mycobiome, bacteriome, inflammation, and diet in people living with HIV
Source: Gut Microbes. 2022 Jun 23;14(1):2089002. doi: 10.1080/19490976.2022.2089002 (PMC9235884; doi:10.1080/19490976.2022.2089002)
Supplement: Supplemental Material [file KGMI_A_2089002_SM7564.zip › SupplementaryMaterials/SupplementaryTables.doc]

**Supplementary Materials:**

**Title:** Interactions among mycobiome, bacteriome, inflammation and diet in people living with HIV

**Authors:** María José Gosalbes, Nuria Jimenéz-Hernandéz, Elena Moreno, Alejandro Artacho, Xavier Pons, Sonia Ruíz-Pérez, Beatriz Navia, Vicente Estrada, Mónica Manzano Alba Talavera-Rodriguez, Nadia Madrid, Alejandro Vallejo, Laura Luna, José A. Pérez-Molina, Santiago Moreno, Sergio Serrano-Villar

**The following supporting information is available for this article:**

**Supplementary Table 1:** Wilcoxon rank-sum test between the relative abundance of the ASVs detected with sPLS-DA in PWH and control (HIV-) groups.

**Supplementary Table 2:** Association index between mycobiome and bacteriome in PWH group.

**Supplementary Table 3:** Association index between mycobiome and bacteriome in control (HIV-) group.

**Supplementary Table 4:** Immune activation markers data. Data are presented as mean.

**Supplementary Table 5:** Association index between microbiome and immune activation markers in the PWH group.

**Supplementary Table 6:** Food consumption and nutrient intake data.

**Supplementary Table 7:** Association index between food consumption and nutrient intake and microbiome in the PWH group.
